# Thalamic gating contributes to forward suppression in the auditory cortex

**DOI:** 10.1371/journal.pone.0236760

**Published:** 2020-07-29

**Authors:** Colin Xiong, Xiuping Liu, Lingzhi Kong, Jun Yan

**Affiliations:** Department of Physiology and Pharmacology, Hotchkiss Brain Institute, Cumming School of Medicine, University of Calgary, Calgary, Alberta, Canada; University of Michigan, UNITED STATES

## Abstract

The neural mechanisms underlying forward suppression in the auditory cortex remain a puzzle. Little attention is paid to thalamic contribution despite the important fact that the thalamus gates upstreaming information to the auditory cortex. This study compared the time courses of forward suppression in the auditory thalamus, thalamocortical inputs and cortex using the two-tone stimulus paradigm. The preceding and succeeding tones were 20-ms long. Their frequency and amplitude were set at the characteristic frequency and 20 dB above the minimum threshold of given neurons, respectively. In the ventral division of the medial geniculate body of the thalamus, we found that the duration of complete forward suppression was about 75 ms and the duration of partial suppression was from 75 ms to about 300 ms after the onset of the preceding tone. We also found that during the partial suppression period, the responses to the succeeding tone were further suppressed in the primary auditory cortex. The forward suppression of thalamocortical field excitatory postsynaptic potentials was between those of thalamic and cortical neurons but much closer to that of thalamic ones. Our results indicate that early suppression in the cortex could result from complete suppression in the thalamus whereas later suppression may involve thalamocortical and intracortical circuitry. This suggests that the complete suppression that occurs in the thalamus provides the cortex with a “silence” window that could potentially benefit cortical processing and/or perception of the information carried by the preceding sound.

## Introduction

Sounds in nature are complex in their physical properties and temporal relations. Many sounds are suppressed or attenuated by the auditory system and consequently one does not hear or comprehend all the sounds that impinge upon the eardrum in short time intervals. Forward masking is a psychoacoustic phenomenon that describes how perception of a sound is affected by a preceding sound [1]. Psychoacoustic studies demonstrate how forward masking is dependent on the correlation of the physical properties (eg., frequency and amplitude) of preceding and succeeding sounds [2–5]. The temporal separation between two sounds appears a critical factor; the masking effect is rarely observed when two sounds are separated by more than ~200 milliseconds [2,5–7].

In concert with this behavioral phenomenon, the neurons in the auditory cortex exhibit no or reduced responses to two sounds that occur in close succession. The suppression period after the preceding stimulus varies from tens to hundreds of milliseconds depending on the physiological property of neurons and the physical property of stimuli [8–14]. The neural mechanism for forward suppression in the auditory cortex requires further investigation. Available evidence tends to favor a complex synergy including intracortical, intrathalamic and GABAergical contributions [9,11,15,16]. From this point of view, the forward suppression in the thalamus is likely an important component of the process leading to cortical suppression. That is, owing to the hierarchical regime of the auditory system, the forward suppression present at lower levels in the system should be transmitted to higher levels.

Of note, forward suppression measured in the two-sound stimulus paradigm already occurs in the auditory periphery; the preceding sound only partially suppresses the responses to the succeeding one [17–21]. The suppression period at the auditory nerve can be up to 150 ms [18]. In the central auditory system, studies in different species of animals show that the suppression period in the two-tone paradigm can be up to ~300 ms in the auditory cortex [9,13,16], up to ~100 ms in the thalamus [21] and about ~150 ms in the midbrain [22]. The shorter suppression period observed in the thalamus but not the midbrain may be due to the use of different animal models and experimental designs. Other studies examine neural adaptation by using repetitive stimulus and manifest a neural phenomenon called rate-following response; auditory neurons are shown to decrease and even to cease their responses to repetitive sound when the repetition rate reaches higher levels. The capacity of the rate-following response is highest in the auditory nerve and lowest in the auditory cortex. This capacity of thalamic neurons appears to fall between those of neurons in the midbrain and cortex [23–28]. Since the temporal interval between sounds is a key factor in both repetitive sound and two-tone stimulus paradigms, we hypothesize that the period of forward suppression in the auditory thalamus may also fall between the suppression periods found in the cortex and midbrain.

To investigate the potential contribution of the thalamus to cortical forward suppression, we compared the time courses of forward suppression between the primary auditory cortex (AI) and the ventral division of the medial geniculate body (MGBv) in C57 mice. We found that the preceding tone led to complete suppression of the neuronal responses to the succeeding tone in both AI and MGBv; the periods of complete suppression were 123.3 ± 43.4 ms and 75.1 ± 59.3 ms, respectively. We also found that the period of complete suppression of thalamocortical field excitatory postsynaptic potentials (fEPSPs) was 107.9 ± 24.6 ms, between the complete suppressions of AI and MGBv neurons.

## Materials and methods

The animals and recordings in the AI and MGBv in this study are mostly the same as those used in our other publications [29,30]. Additional details are described below. The mouse protocol was approved by the Animal Care Committee at the University of Calgary (protocol number: M04044).

### Animal preparation

Twenty-one female C57 mice aged 4–7 weeks and weighing 14.6–20.7 g, were used in this study. Animals were anesthetized using an intraperitoneal injection of ketamine (85 mg/kg, Bimeda-MTC Animal Health Inc., Canada) and xylazine (15 mg/kg, Bimeda-MTC Animal Health Inc.). The anesthetic level was maintained by additional doses of ketamine and xylazine (17 mg/kg and 3 mg/kg). Under anesthesia, the mouse’s head was fixed in a custom-made head holder by clamping between the palate and nasal/frontal bones. The scalp was then incised along the midline and subcutaneous tissues/muscle were removed to expose the skull including the bregma and lambda. A mouth bar was adjusted to align the bregma and lambda of the skull at the same level. Once the mouse’s head was positioned, two holes (~2 mm in diameter) were drilled to expose the left parietal cortex above the medial geniculate body of the thalamus (3.1 mm posterior to bregma, 1.8–2.1 mm lateral to the midline) and the left auditory cortex (2.2–3.6 mm posterior to bregma, 4–4.5 mm lateral to the midline). After surgery, the animal was placed in a soundproof chamber. Throughout the course of surgery and electrophysiological experiments, the animal’s body temperature was maintained at a constant 37°C using a feedback-controlled heating pad.

### Acoustical stimulation

Pure tones of 20-ms duration with 5-ms rise and fall times were used for acoustic stimuli. The tones were digitally synthesized and converted into analog sinusoidal waves using an Enhanced Real-time Processor (RP2, Tucker-Davis Tech., Gainesville, FL, USA). These signals were then fed to a free-field loudspeaker (ES1, Tucker-Davis Tech., Gainesville, FL, USA) via a digital attenuator (PA5, Tucker-Davis Tech., Gainesville, FL, USA). The loudspeaker was placed 45 degrees to the right of and 13 cm away from the mouse’s right ear. Its output was calibrated at the right ear of the animal with a condenser microphone (Model 2520, Larson-Davis Laboratories, USA) and a microphone preamplifier (Model 2200C, Larson-Davis Laboratories, USA), and tone intensity was expressed as dB SPL (re. 20 μPa). Frequencies and intensities of tone were varied either manually or automatically using BrainWare data acquisition software. (Tucker-Davis Tech., Gainesville, FL, USA).

### Recording in the AI

**Fig 1:**
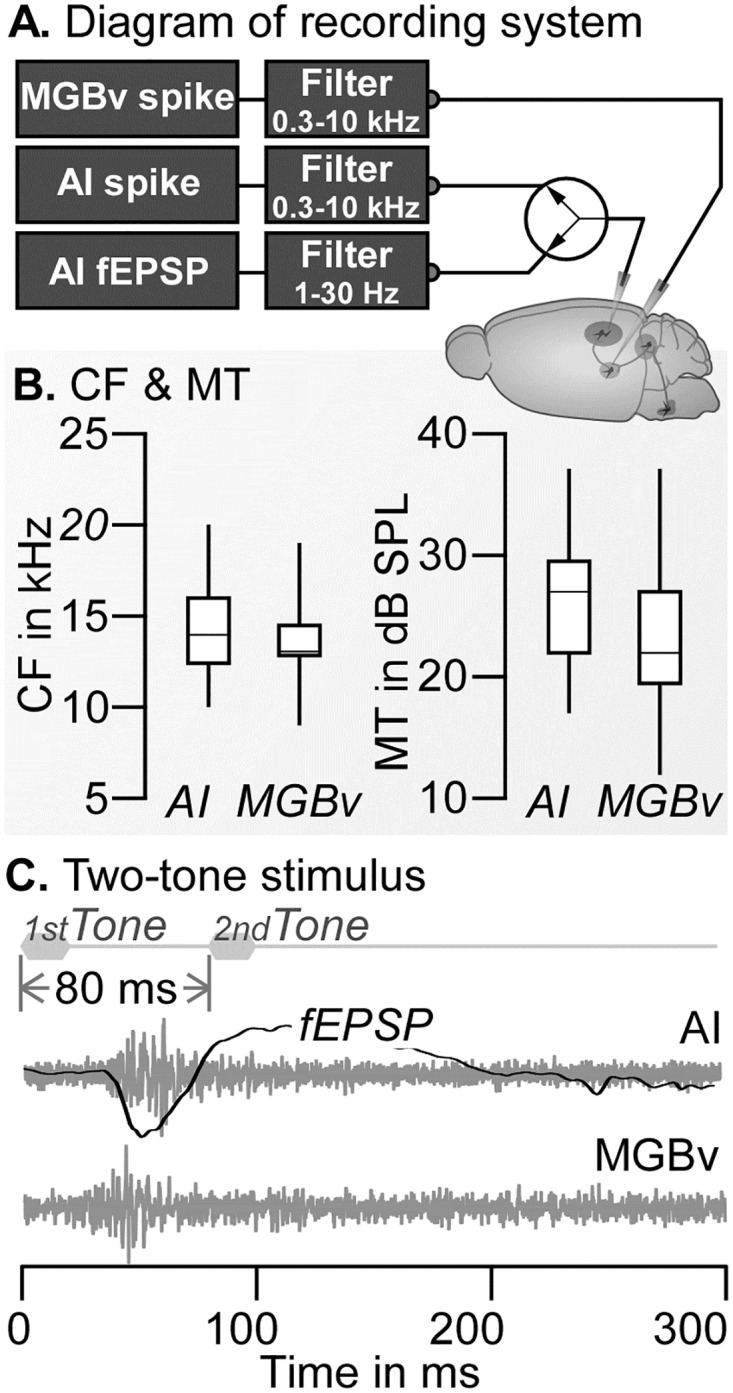
Diagram of the recording system (A), CFs and MTs of recorded neurons (B) and examples of original traces recorded from AI and MGBv (C). The 1^st^-tone and 2^nd^-tone were physically identical but temporally separated by 80 ms. The original traces began at the onset of the 1^st^ tone. For both AI and MGBv neurons, the 1^st^-tone evoked robust response. The 2^nd^-tone evoked no visible response.

A tungsten electrode with tip impedance of ~2 MΩ was perpendicularly penetrated into the left auditory cortex. Bioelectrical signals from an electrode were fed to the PA16 preamplifier and RZ6 amplifier (Tucker–Davis Tech., Gainesville, FL, USA). Bioelectrical signals were divided into two recording channels. One channel measured spikes amplified 10,000 times, and filtered by a bandpass of 0.3–10 kHz. The other channel measured the cortical local field potential, ie., thalamocortical field excitatory postsynaptic potential (fEPSP [31]) amplified 1,000 times and filtered by a bandpass of 1–30 kHz (Fig 1A). All bioelectrical signals were digitized at a sampling rate of 25 kHz and were stored using BrainWare acquisition software. Tone-evoked responses were typically recorded at a depth of 400–700 μm below the brain surface. Three to eight penetrations were required to determine the AI based on a cortical tonotopic map, ie., the characteristic frequencies (CF) of the tested cortical neurons.

### Recording in the MGBv

A tungsten electrode with a tip impedance of ~2 MΩ was vertically penetrated to the left thalamus. The electrode was connected to the RA-16 preamplifier of the recording system. The bioelectrical signals were fed to the third channel of the RA-16 preamplifier, amplified 10,000 times and filtered by a bandpass of 0.3–10 kHz (Fig 1A). The tone-evoked responses were typically observed at a depth of about 3.5 mm below the brain surface. When the neurons showed reduced responses to 1-Hz repetitive tones and had broad frequency tuning, the electrode was withdrawn and repositioned at left-right and anteroposterior placements. Otherwise, the electrode was further advanced until the tone-evoked responses disappeared and then the electrode was withdrawn a distance of 50–100 μm; these were credible measures to ensure the electrode tip in the MGBv according to our histological confirmation [29,30].

### Sampling receptive field of AI and MGBv neurons

Once the electrodes were placed in the AI and MGBv, tones were delivered at a rate of 1/s. The bioelectrical signals, delivered in a window of 200 ms from tone onset, were measured using BrainWare data acquisition software. Tone frequency and amplitude were randomly varied (FA-scan); the frequency ranged from 3 kHz to 40 kHz using a 1 kHz increment and the amplitude ranged from 87 dB SPL to minus -13 dB SPL in 5 dB increments. A complete set of stimuli consisting of 798 different tones or recording blocks were delivered as FA-scan. An identical FA-scan was repeated 5 times to sample a reliable excitatory area. The receptive field was defined as the frequency-amplitude area encompassing the excitatory responses of given neurons. The receptive fields of AI and MGBv neurons were sampled concurrently. The entire course required ~14 minutes.

### Two-tone stimulus

The classical two-tone stimulus paradigm was employed to examine the forward suppression in the AI and MGBv, including the output (spikes) of MGBv neurons and input (fEPSP)/output (spikes) of AI neurons. Since this study focused on the time course and considering that the two-tone suppression is sensitive to the physical difference between two tones, the preceding and succeeding tones were designed to be identical; tone frequency was set at the CF and tone amplitude was set at 20 dB above the minimum threshold of given neurons. The forward suppression was tested for 17 temporal intervals between the preceding and succeeding tones (in milliseconds from onset to onset). The succeeding tone was delayed by 510 ms from the trial onset and the preceding tone had random delays from the trial onset (20 ms to 500 ms with 30 ms step) from trial to trial. Therefore, the temporal separation of the succeeding tone from the preceding tone ranged from 10 ms to 490 ms with 30 ms increments. For simplicity, we term this the inter-stimulus interval (ISI) and the procedure is described as an ISI-scan. An ISI-scan had 17 trials and each trial was 1000-ms in length. An identical ISI-scan was repeated 10 times. The bioelectrical signals in the first 700 ms of each trial were recorded using BrainWare data acquisition software.

### Data processing

Original bioelectrical signals for the AI spikes, AI fEPSPs and MGBv spikes were stored in different DAM files by BrainWare data acquisition software. Data were read and processed using our custom-made data processing software named SoundCode. To extract the spike information, the average amplitude of background voltage fluctuation was first calculated and a trigger level of 20% greater than the averaged background amplitude was determined to detect neuronal firings, so-called spikes that could be either spontaneous or stimulus-evoked. Spikes (multiple unit) were sorted based on eight parameters of the spike waveform [29,32,33]. The 20% trigger level together with spike sorting were designed to ensure the maximal exclusion of background noise. The pattern of neuronal firing (multiple units) was displayed as raster, post-stimulus histogram (PST) or accumulative PST (PSTC). To extract fEPSP information, 10 trials to an identical stimulus were first averaged and the background fluctuation was then calculated from the averaged trial without a tone-evoked event, ie., prior to either preceding or succeeding tone stimulus. Thalamocortical fEPSP was determined when a negative-going wave was 20% greater than the background fluctuation. Finally, the following measurements were made to characterize the stimulus-evoked responses.

1. *Spike number*: This was the sum of spikes to identical stimuli within a window of 10–60 ms from the stimulus onset. The window was adjusted in some cases such as those involving extremely long latency latencies and/or response durations.
2. *Spike response latency*: This was measured based on the PSTC. The response latency was the time interval from the onset of tone to the intersection point of the baseline and the rising slope of the PSTC.
3. *fEPSP amplitude*: The fEPSP amplitude was defined as the microvolt difference between the baseline and the negative-going peak.
4. *fEPSPs latency*: This was the time interval from tone onset to the intersection point of the baseline and the rising slope of the waveform.
5. *Characteristic frequency (CF) and Minimum threshold (MT)*: The CF and MT were determined based on multiunit receptive fields of AI and MGBv neurons. The CF was the frequency to which given neurons exhibited the lowest response threshold. The MT was the lowest response threshold of given neurons across frequencies.
6. *Forward modulation (Q*_*R*_*)*: This was the quotient or ratio of the responses to the succeeding stimulus. Specifically, the response magnitude (spike number or fEPSP amplitude) to the succeeding tone was divided by those to the preceding tone. Q_R_ = 0 indicates complete suppression. Q_R_ = 0.5 indicates 50% suppression and Q_R_ = > 1 indicates no suppression or facilitation.

### Statistical processing

Data was presented as mean ± SD. The *ANOVA* test was used to compare the differences between groups of data and numbers. A *p* value of less than 0.05 was statistically significant.

## Results

Only one data set were collected from each mouse. Since cortical recordings were not possible in 3 of the 21 mice, we collected 18 AI data (spike and fEPSP) and 21 MGBv data (spike) in total. The receptive fields of both AI and MGBv neurons were sampled by an FA-scan to determine the tone parameters for two-tone stimulus, ie., CFs and 20 dB above the MTs. The ranges and medians of CFs and MTs are shown by Box Plot in Fig 1B. On average, the CF and MT of AI neurons were 14.67 ± 2.69 kHz and 25.89 ± 5.67 dB SPL (n = 18). The CF and MT of MGBv neurons were 13.86 ± 2.36 kHz and 23.91 ± 6.98 dB SPL (n = 21). The CFs and MTs of sampled AI and MGBv neurons were within the central hearing range of C57 mice [34,35]. The response latency to tone at CF and 20 dB above MT was 23.3 ± 8.1 ms for AI neurons, 17.3 ± 5.7 ms for MGBv neurons and 21.8 ± 5.5 ms for AI fEPSP.

Fig 1C illustrates the typical effects of two-tone forward suppression in the AI and MGBv. Both AI and MGBv sites showed identical CF (14 kHz) and MT (22 dB SPL). A tone of 14 kHz and 42 dB SPL induced robust responses (spikes) in both neurons with a shorter latency exhibited by the MGBv neurons. When the same tone was delivered again with an 80-ms delay, these two neurons showed no identifiable responses. The AI fEPSP was also evoked by the first tone but not by the second tone. This example clearly demonstrated strong auditory forward suppression in both the AI and MGBv.

**Fig 2:**
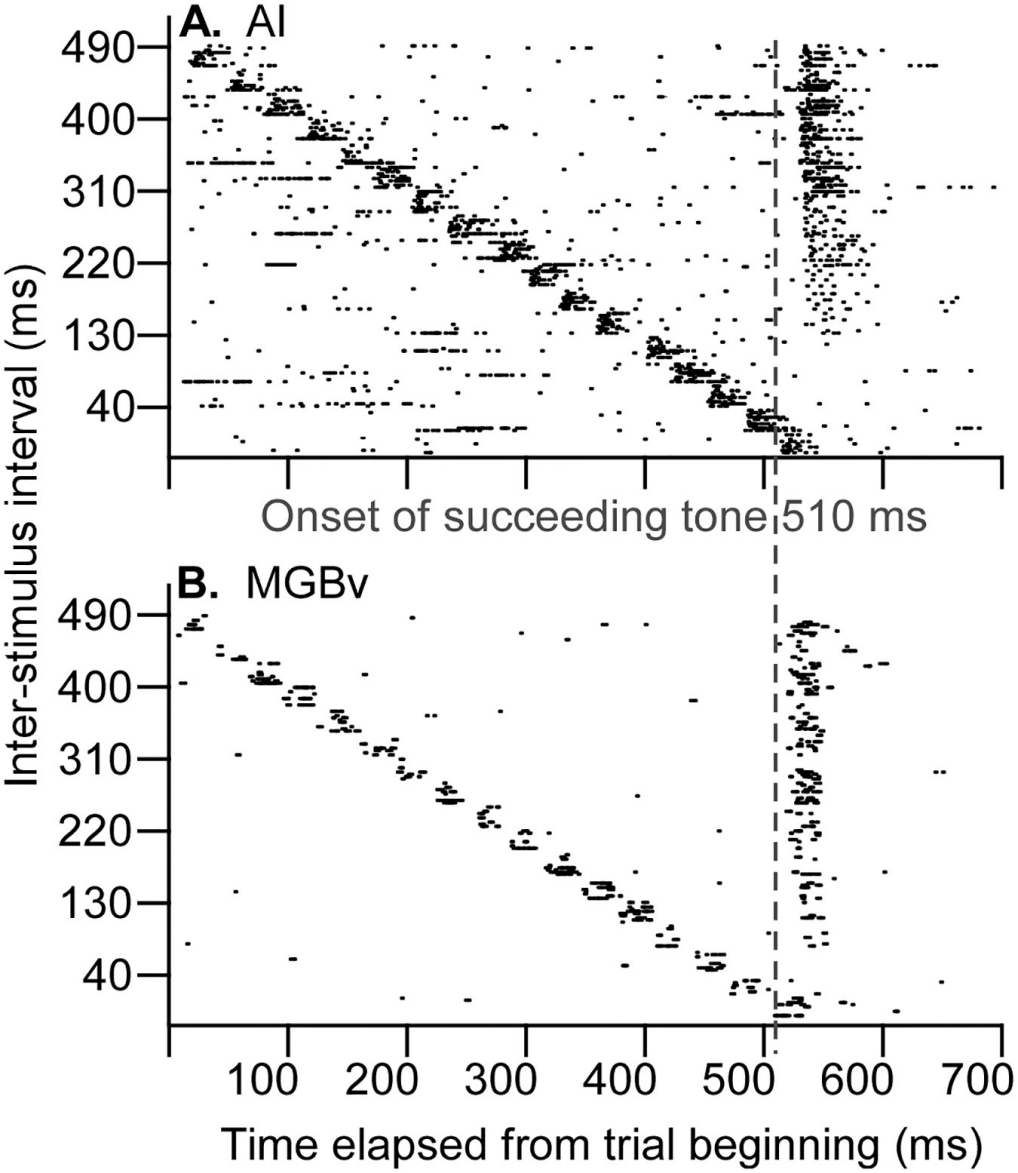
Raster plotting of spikes from trial to trial. The responses to preceding tone are pronounced and mostly consistent, and those to the succeeding tone decreased following the decrease in the ISI between preceding and succeeding tones in both the AI (**A**) and MGBv (**B**) neurons. The responses to succeeding tone ceased at 130-ms ISI (or shorter ISIs) in the AI neuron and 70-ms ISI (or shorter ISIs) in the MGBv neuron. Dashed lines indicate the onset of succeeding tones.

To compare the time course of the forward suppression between AI and MGBv neurons, the neuronal responses to two-tone stimuli with various ISIs were examined. The examples shown in Fig 2 illustrates typical responses to an ISI-scan of AI and MGBv neurons. The spikes of both AI and MGBv neurons to the preceding tones (various onsets) were mostly consistent whereas those to the succeeding tone (identical onset) gradually decreased following the decrease in the ISI of two tones although the ISIs were randomly varied. These two sets of data were found to be typical and confirmed several observations of two-tone forward suppression. First, complete suppression occurred in both AI (Fig 2A) and MGBv (Fig 2B) neurons when the ISIs were sufficiently short. Second, the response latencies were lengthened along with the decrease in spike numbers or ISIs in both AI and MGBv neurons during partial suppression. Third, the forward suppression appeared to be stronger for the AI neurons than the MGBv neurons when ISIs were less than 300 ms. Finally, complete suppression occurred in the AI neurons when the ISI was 130 ms or less and in the MGBv neurons when the ISI was 70 ms or less.

**Fig 3:**
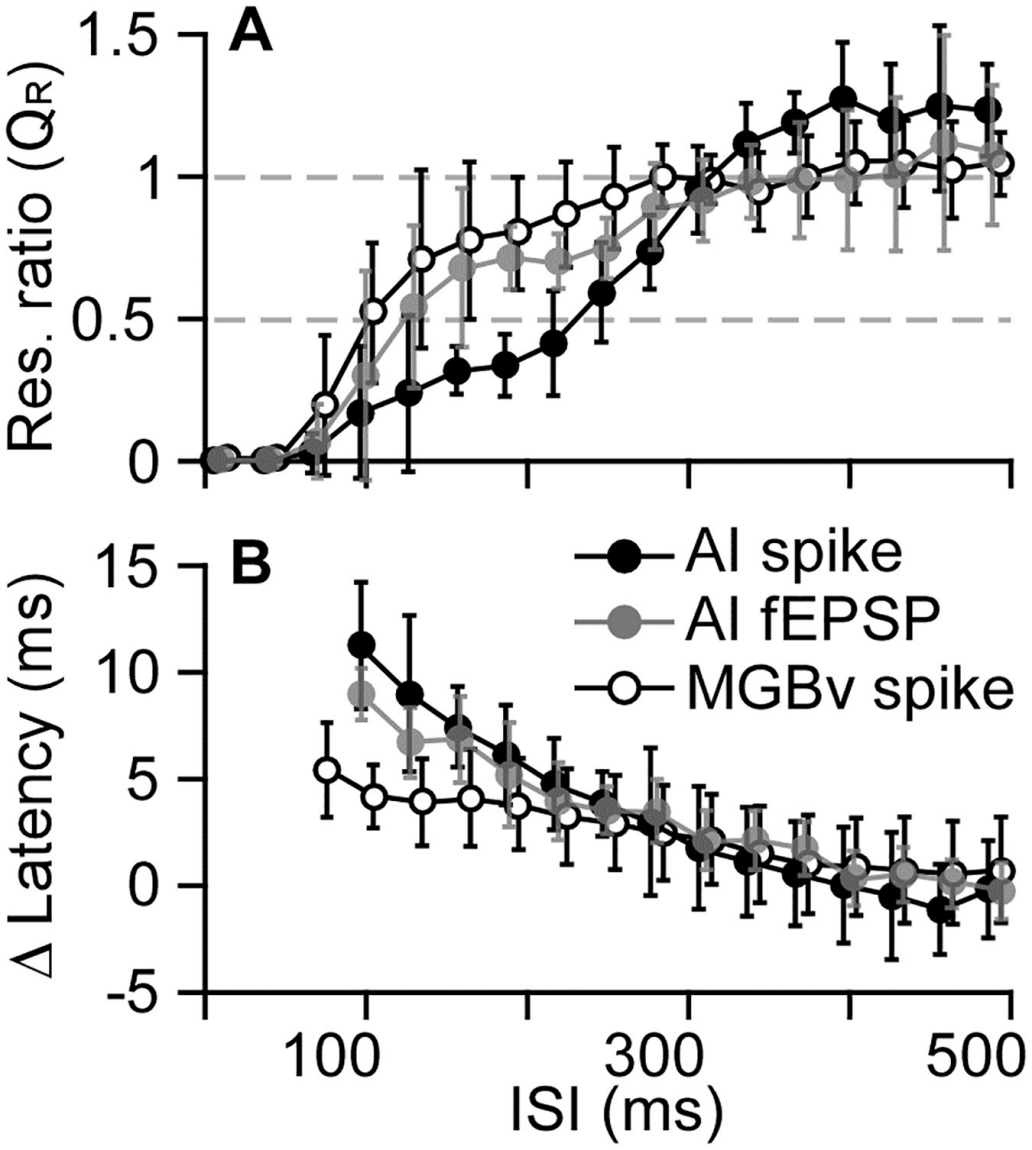
Changes in AI spikes, AI fEPSPs and MGBv spikes (A) and changes in their latencies (B) in response to succeeding tone were plotted as the function of the ISI.

These observations were further illustrated and confirmed by the averaged changes in spikes and latencies as the function of the ISI (Fig 3). In general, the shorter the ISI, the lower the Q_R_ (response magnitude to succeeding tone divided by that to preceding tone) and the longer the latency. The neuronal responses to succeeding tone were completely suppressed (Q_R_ = 0) when the ISI was < 40 ms for both AI and MGBv neurons. At an ISI of 70 ms, the complete suppression of the responses to succeeding tone was not observed in 5 out of 18 (27.8%) AI recording sites and 16 out of 21 (76.2%) MGBv recording sites. Starting from an ISI of 70, the suppression of the responses to succeeding tone gradually decreased (Q_R_ increased). The Q_R_ increases were much slower for AI neurons than for MGBv neurons (curve shift is towards the right). The responses to succeeding tone were suppressed by 50% (Q_R_ = 0.5) when the ISI was 220 ms in the AI as compared to 100 ms in the MGBv (Fig 3A, black). Suppression was not observed in either the AI or the MGBv when the ISI was equal to or larger than 310 ms. In concert with the decrease in spikes to succeeding tone, the response latency gradually increased following the shortening of ISI (Fig 3B, black). That the change in the latency was greater in the AI than in the MGBv was clearly demonstrated.

**Fig 4:**
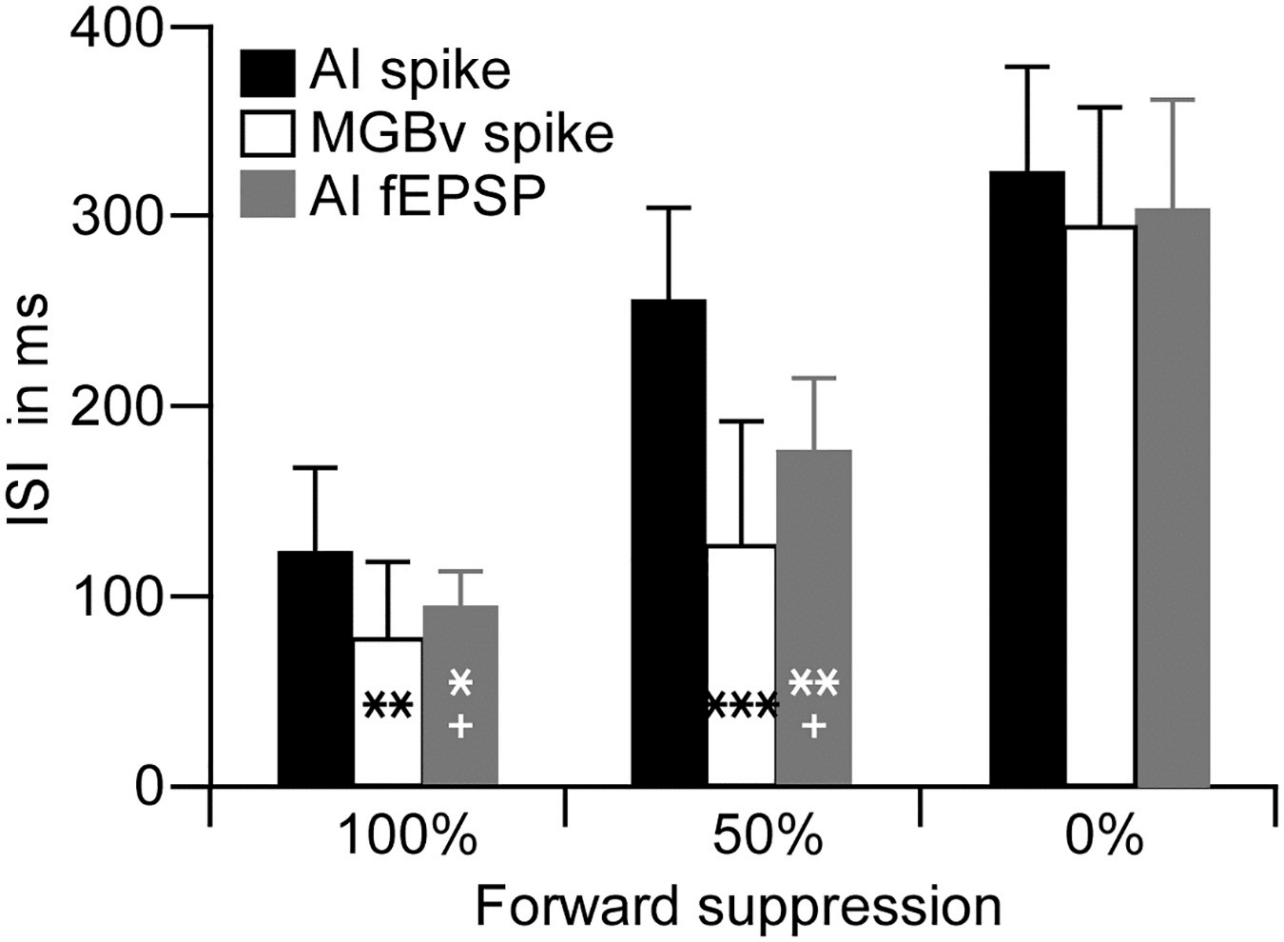
Comparison of the ISIs that caused 0%, 50% and 100% decreases in response magnitudes (AI spikes, AI fEPSP and MGBv spikes) to succeeding tone. **: p < 0.01; *** < 0.001, as compared with the AI spike. +: p < 0.05, as compared with the MGBv spike.

To quantify the suppression periods, the ISIs that induced 100% suppression (complete suppression), 50% suppression (partial suppression) and 0% suppression (no suppression) in the AI and MGBv were calculated and are shown in Fig 4. The averages of the 100% suppression periods were 123.3 ± 43.4 ms (n = 18) for AI neurons and 75.1 ± 59.3 ms (n = 21) for MGBv neurons. These averages were significantly different (p < 0.01). The averaged periods of the 50% suppression were 251.7 ± 44.0 ms (n = 18) for AI neurons and 128.9 ± 67.2 ms (n = 21) for MGBv neurons, which were also significantly different (p < 0.001). The averages of entire suppression periods were 323.3 ± 59.0 ms (n = 18) for AI neurons and 294.6 ± 63.5 ms (n = 21) for MGBv neurons, which had no statistical significance (p >0.05).

**Fig 5:**
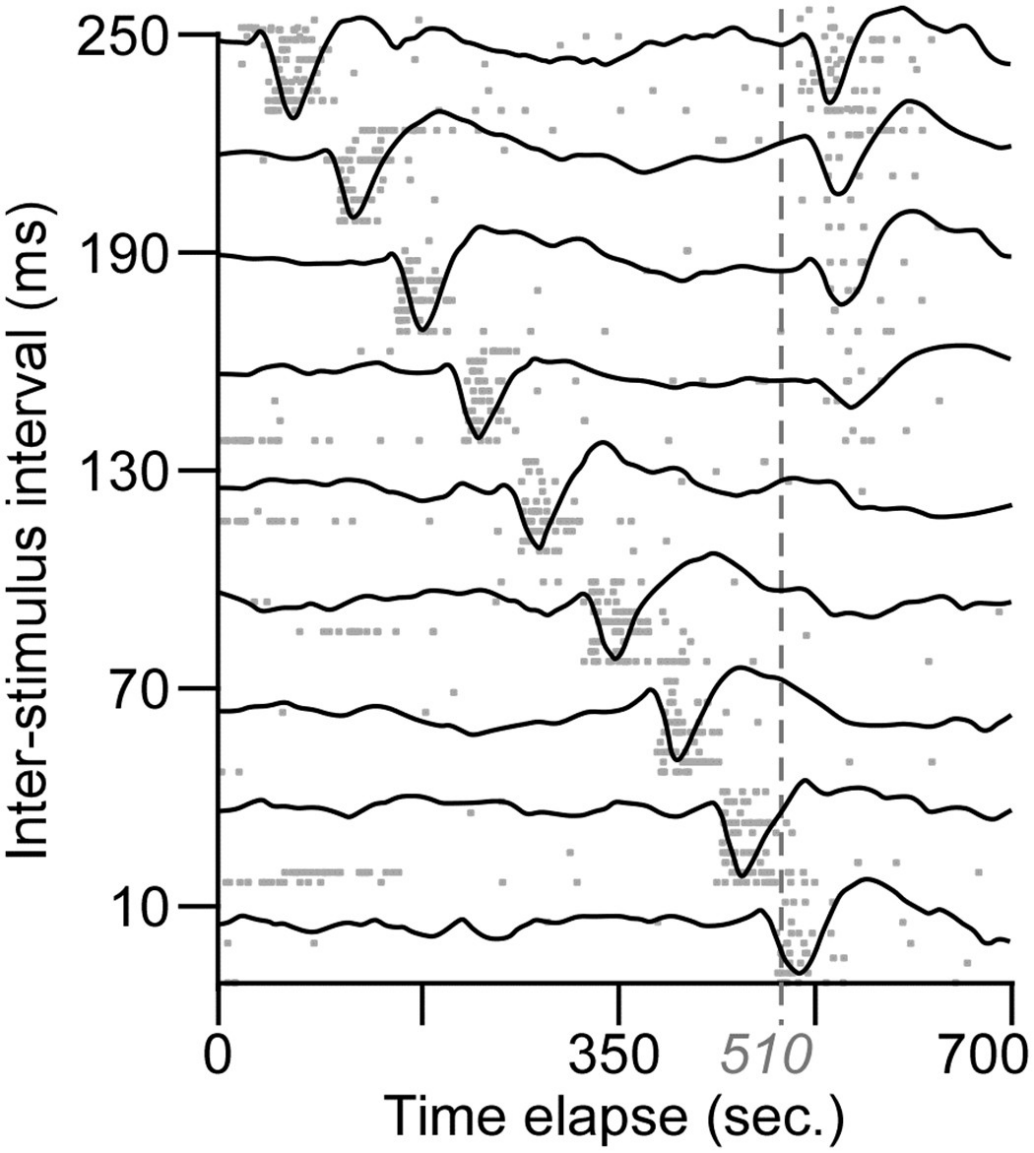
Example of AI fEPSPs superimposed on AI spikes in response to two-tone pairs with various ISIs.

Owing to the determinant role of the thalamocortical system in cortical function [30,36], the significant difference in the period of forward suppression in the AI and MGBv raised the question of how the AI fEPSPs to succeeding tone were modulated or suppressed as the function of various ISIs. The AI fEPSPs were sampled together with spikes of AI neurons by using the same electrode but different frequency filters (Fig 1A). A typical example is illustrated in Fig 5 The fEPSP evoked by succeeding tone gradually decreased in amplitude and was not discernable when the ISI was equal to or shorter than 130 ms. On average, the forward suppression of AI fEPSP emerged between the suppressions of AI and MGBv spikes but closer to the MGBv suppression (Fig 3A, gray). The Q_R_ of AI fEPSPs were mostly between AI and MGBv Q_R_s when the ISIs was between 70 and 310 ms. In other words, the suppression of AI fEPSP magnitude was weaker than that of the AI spikes but stronger than that of the MGBv spikes (Fig 4). The change in response latency of AI fEPSP was smaller than that of the AI spike and larger than that of the MGBv spike (Fig 3B).

As with the analysis of AI and MGBv spikes in response to the temporal separation, the ISIs characterized by 100% suppression, 50% suppression and 0% suppression were measured for AI fEPSPs. On average, the ISI required for 100% suppression was 107.9 ± 24.6 ms in AI fEPSP. This suppression window was significantly shorter than that of the AI spikes (p < 0.05) whereas significantly longer than that of the MGBv spikes (p < 0.05). The ISI of 50% suppression of AI fEPSPs was 185.6 ± 31.6 ms, which was also significantly longer than the MGBv spikes (p < 0.05) and shorter than the AI spikes (p < 0.01). The entire suppression period (ISI for 0% suppression) of AI fEPSP was 316.7 ± 57.0 ms, which was between those of AI and MGBv spikes; there were no statistical significance among them (p > 0.05).

## Discussion

Forward suppression, a physiological phenomenon of auditory neurons across species, is a fundamental property contributing to auditory scene analysis [37–39]. Previous studies demonstrate that forward suppression in the auditory cortex is associated with the physical properties of preceding and succeeding stimuli. For example, the more efficacious suppression is often observed when the frequencies of two tones are similar [9,10,12,13,40]. Two lines of evidences suggest that the temporal separation between two stimuli is probably a more important factor for forward suppression. One proposes that auditory neurons show adaptation to sound sequences or amplitude-modulated sounds having a high repetition rate [41–43]. The other proposes that the preceding tone can suppress the responses of given neurons to succeeding tone even if two tones are different in frequency and amplitude provided that the interval between two tones is sufficiently short [9].

As forward suppression is proposed to increase gradually along the ascending auditory pathway, the period of the suppression caused by preceding stimulus could be shorter in the thalamus than in the auditory cortex as shown in work involving repetitive sound stimulation [22,24,28,42,44]. Studies of stimulus-specific adaptation using the oddball stimulus paradigm demonstrate that the deviance detection increases from the auditory midbrain to thalamus to cortex [45–48]. Based on use of the two-tone stimulus paradigm, our data demonstrate that the responses of both AI and MGBv neurons to the succeeding tone reached the responses evoked by the preceding tone (Q_R_ = ~1) when the ISIs were ~300 ms or longer (Figs 3A and 4). This suggests that the overall suppression periods were similar in the auditory thalamus and cortex. The 300-ms suppression period in the auditory cortex of C57 mice is comparable to those using the two-tone paradigm in other species [9,13,16,40]. However, the periods of complete and partial suppression were clearly different between the AI and MGBv; the periods of 50% and 100% suppression of the responses to succeeding tone were significantly longer in the AI than in the MGBv (Figs 2, 3A and 4). According to the time course, the forward suppression in the MGBv appeared to have two distinct periods (Fig 3). One is a period of complete suppression–the ISI from preceding onset to 75.1 ± 59.3 ms (Q_R_ = 0) and the other, a period of partial suppression–the ISI from 75.1 ± 59.3 ms to ~300 ms (0 < Q_R_ < 1). These two suppression periods in the MGBv provide us with valuable information regarding our understanding of the neural mechanisms for forward suppression in the auditory cortex. The mechanism must consist of at least two components. One is thalamic forward suppression and the other is some combination of thalamocortical and cortical circuitry.

The concept of neural inhibition is also viewed as a factor underlying neural suppression in the auditory cortex [9,49]. Studies in this area involve direct measurement of the membrane properties of cortical neurons and different types of cortical interneurons; inhibition is dependent on cell types and is found to take effect shortly after the preceding stimulus in two-sound stimulus paradigm [11,15,16]. The inhibitory conductances of cortical neurons to preceding tone rarely last longer than 50–100 ms [16]. Our MGBv data indicate that these mechanisms may not be required during the period of complete suppression in the MGBv, at least when the preceding and succeeding tone are physically identical. The occurrence of complete suppression means that MGBv neurons and subsequently their thalamocortical fibers did not discharge in response to the succeeding stimulus. In other words, cortical neurons did not receive upcoming synaptic inputs associated with the succeeding stimulus physically identical to that of the preceding one. This favors previous findings that cortical application of the GABA_A_ and GABA_B_ receptor antagonists does not prevent the occurrence of forward suppression [50]. However, this does not necessarily mean that the contribution of cortical inhibition to the forward suppression in the auditory cortex can be excluded [11,12,16].

The complete suppression period in the MGBv also implies that the target neurons in the AI acquire a “silence” window of 75.1 ± 59.3 ms after receiving the preceding thalamocortical inputs. This silence window must enable the gating of subsequent upcoming signals to given cortical neurons for ~75 ms. It is worth noting here that this “silence” window can be longer or shorter depending on the physical difference in the preceding and succeeding stimuli (eg., frequency, amplitude, duration) [2,3,9,10,12]. This “silence” window suggests that the targeted cortical neurons and their associated neural circuity acquire a noise-free window for processing and/or consolidating the information carried by the preceding sound. A noise-free environment can be essential and critical for neural circuits to implement complex computations without interference, such as the oscillation in thalamocortical circuitry [51–54]. It is interesting that the “silence” window (window of thalamic suppression) appears close to the duration of thalamocortical excitatory postsynaptic events (EPSP) and the field EPSP evoked by tone or thalamic stimulation (Fig 3) [16,30,31,55]. An important and currently unrecognized mechanism for cortical information processing and perception might be responsible for this equivalence if this is not a coincidence. In any case, the biological significance of the silence window warrants further investigation.

During the period of partial suppression (75.1 ± 59.3 ms to ~300 ms from the onset of the preceding tone), the decrease in the succeeding responses of AI neurons was significantly larger than that of MGBv neurons; the suppression function in A1 is shifted towards a longer ISI. At 50% suppression, the ISI difference was as large as approximately 120 ms (Fig 3A). Undoubtedly, the AI neurons received weaker succeeding than preceding inputs from the MGBv at each ISI during this period. The additional suppression in AI must hinge on thalamocortical and cortical mechanisms.

A number of mechanisms that cause the forward suppression have been proposed, including vesicular pool depletion and postsynaptic desensitization [56], prolonged refractory periods of postsynaptic potential like after-hyperpolarization [38,57], and inhibition circuity like GABAergic inhibitions [8,9]. These mechanisms however, are not definitive. As for the mechanisms for cortical forward suppression, recent studies indicate the importance of thalamocortical projections [11] and cortical microcircuitry [15,16]. Our fEPSP data suggest that the thalamocortical synaptic mechanism is likely less important than intracortical mechanism such as cortical lateral and feedforward circuits [1,58,59] because the strength of the forward suppression of AI fEPSP was closer to the suppression of the MGBv spike (Fig 3A).

In sum, our data allow us to conclude that forward suppression in the auditory cortex may be exclusively attributed to the suppression period in the thalamus for the first ~75 ms and largely to the intracortical mechanism for the 75–300 ms from the onset of the preceding stimulus. In this species of mice, forward suppression was not exhibited after 300 ms from the onset of the preceding sound in both the thalamus and cortex.
